# Supplementary material for: Bipolar anodal septal pacing with direct LBB capture preserves physiological ventricular activation better than unipolar left bundle branch pacing
Source: Front Cardiovasc Med. 2023 Mar 22;10:1140988. doi: 10.3389/fcvm.2023.1140988 (PMC10073552; doi:10.3389/fcvm.2023.1140988)
Supplement: Supplementary file 1 [file Table1.docx]

**Supplementary Table 1:** A comparison of patients with nsLBBP transitioning to aLBBP with the r/R morphology to those transitioning to aLBBP without the r/R morphology in V1.

|  | nsLBBp transitioning to aLBBP with late r/R morphology in V1  n = 41 | nsLBBp transitioning to aLBBP without the r/R morphology in V1  n = 22 | p value |
| --- | --- | --- | --- |
| Age (years), mean ± SD | 78 ± 10 | 79 ± 6 | 0.54 |
| Male gender, n (%) | 23 (56) | 10 (45) | 0.42 |
| Comorbidities: |  |  |  |
| - Heart failure, n (%) | 5 (12) | 1 (5) | 0.32 |
| - Coronary heart disease, n (%) | 8 (20) | 7 (32) | 0.36 |
| - Diabetes mellitus, n (%) | 10 (24) | 8 (36) | 0.31 |
| - Hypertension, n (%) | 31 (76) | 20 (91) | 0.12 |
| LV ejection fraction (%), mean ± SD | 59 ± 5 | 58 ± 4 | 0.52 |
| Septal thickness, mm, mean ± SD | 10.8 ± 1 | 11.0 ± 1 | 0.41 |
| LVEDD, mm, mean ± SD | 49 ± 6 | 49 ± 5 | 0.80 |
| RVEDD, mm, mean ± SD | 29 ± 4 | 29 ± 5 | 0.82 |
| Pacing indications: |  |  |  |
| - AV block, n (%) | 24 (59) | 11 (50) | 0.59 |
| - SSSy, n (%) | 11 (27) | 10 (45) | 0.12 |
| - Bi-, trifascicular block, n (%) | 3 (7) | 1 (5) | 0.67 |
| - Atrial fibrillation with planned AV junctional ablation, n (%) | 3 (7) | 0 (0) | NA |
| QRS morphology |  |  |  |
| - LBBB, n (%) | 4 (10) | 2 (9) | 0.93 |
| - RBBB, n (%) | 9 (22) | 5 (23) | 0.94 |
| - IVCD, n (%) | 5 (12) | 3 (14) | 0.87 |
| - Narrow QRS, n (%) | 23 (56) | 12 (54) | 0.91 |
| e-DYS, ms, mean ± SD | -23 | -25 | 0.73 |
| QRSd, ms, mean ± SD | 139 | 141 | 0.56 |
| V5RWPT, ms, mean ± SD | 66 | 69 | 0.34 |

LVEDD – left ventricular end-diastolic diameter, LVESD – left ventricular end-systolic diameter, all other abbreviations were previously used in the text.
