# Supplementary material for: Bipolar anodal septal pacing with direct LBB capture preserves physiological ventricular activation better than unipolar left bundle branch pacing
Source: Front Cardiovasc Med. 2023 Mar 22;10:1140988. doi: 10.3389/fcvm.2023.1140988 (PMC10073552; doi:10.3389/fcvm.2023.1140988)

**Supplementary Figure 1:** Panel A: QRSd, e-DYS (Panel B), V5RWPT (Panel C), delay of V1-V8 local activations from the pacing artifact (Panel D), local activations in V1-V8 (first activated segment was placed at 0 ms) (Panel E) and in Panel F local depolarization durations in V1–V8 between nsLBBP transitioning to aLBBP with late r/R and nsLBBP transitioning to aLBBP without the r/R morphology in the V1.


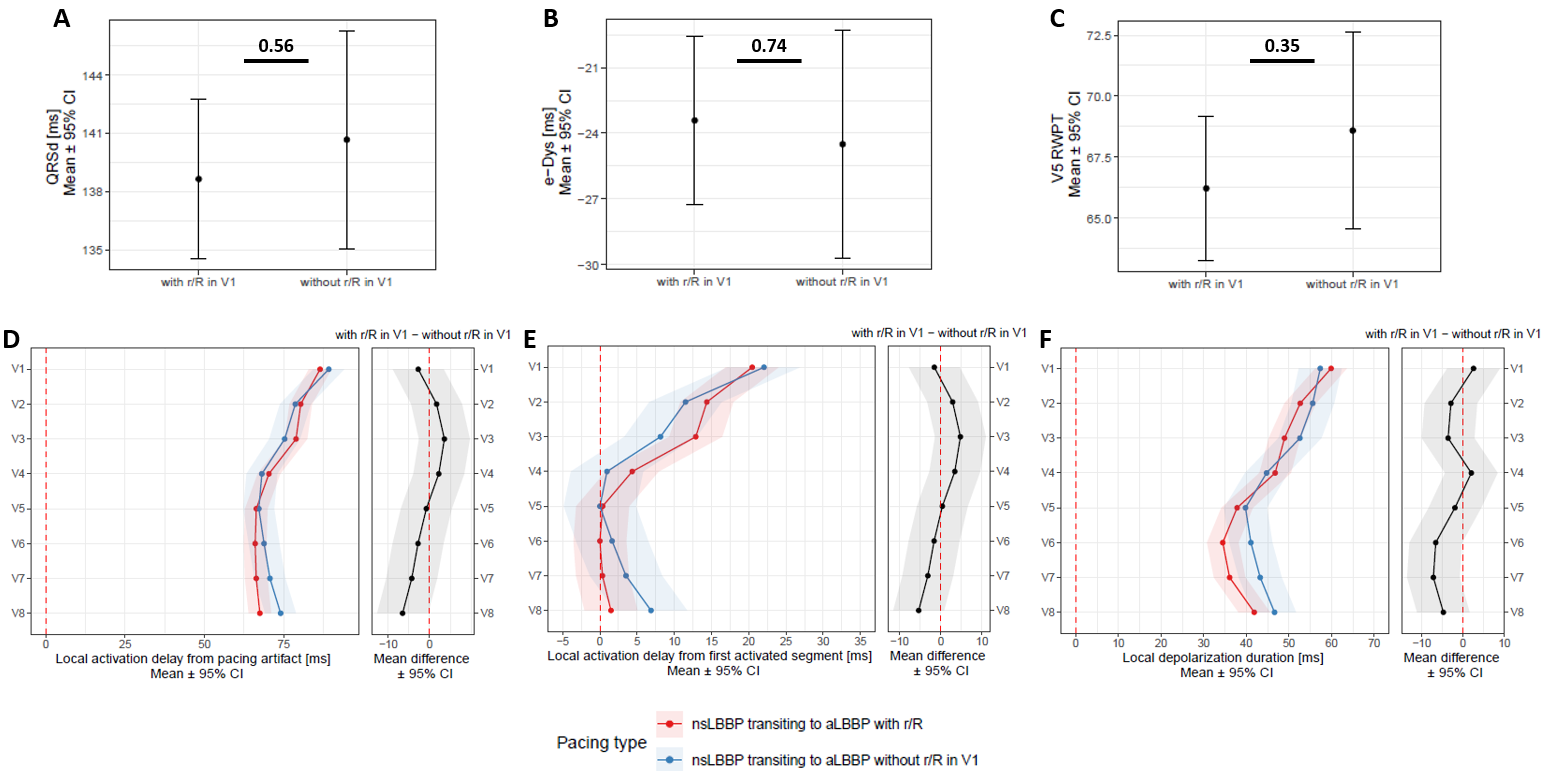

Supplement: Supplementary file 2 [file Datasheet1.docx]
